# Comparison of Hemodynamic Effects of Remimazolam and Midazolam During Anesthesia Induction in Patients Undergoing Cardiovascular Surgery: A Single-Center Retrospective and Exploratory Study

**DOI:** 10.7759/cureus.72032

**Published:** 2024-10-21

**Authors:** Ryosuke Shintani, Taiga Ichinomiya, Keiko Tashiro, Yuri Miyazaki, Tatsuhito Tanaka, Shohei Kaneko, Naoya Iwasaki, Motohiro Sekino, Takuji Maekawa, Tetsuya Hara

**Affiliations:** 1 Department of Anesthesiology and Intensive Care Medicine, Nagasaki University Graduate School of Biomedical Sciences, Nagasaki, JPN; 2 Department of Anesthesiology, Sasebo City General Hospital, Sasebo, JPN

**Keywords:** cardiac output monitoring, cardiovascular surgery, hemodynamic effects, midazolam, remimazolam

## Abstract

Introduction: Patients undergoing cardiovascular surgery may experience hemodynamic instability during the induction of general anesthesia, and anesthetic agents with minimal hemodynamic effects should be administered. Midazolam, a classic benzodiazepine anesthetic, is known to have relatively weak circulatory depression during anesthesia induction compared to other sedatives. On the other hand, remimazolam, a newly approved short-acting benzodiazepine anesthetic, is expected to have fewer circulatory depressant effects. However, comparisons between remimazolam and midazolam regarding circulatory depression during anesthesia induction have not been adequately studied.

Objective: This study aims to compare the hemodynamic effects of remimazolam and midazolam during anesthesia induction in patients undergoing cardiovascular surgery.

Method: In this single-center retrospective and exploratory study, adults undergoing cardiovascular surgery under general anesthesia were divided into the remimazolam group (R group) and midazolam group (M group). Remimazolam 0.06 mg/kg (R group) or midazolam 0.03 mg/kg (M group) was administered during induction of general anesthesia. Both groups received remifentanil 1 μg/kg/min as analgesia. During anesthesia induction, additional sedatives (remimazolam or midazolam, respectively) were administered as needed to maintain the bispectral index (BIS) below 60. The primary endpoints were the following hemodynamic parameters: mean arterial pressure (MAP), heart rate (HR), cardiac index (CI), stroke volume index (SVI), systemic vascular resistance index (SVRI), and stroke volume variation (SVV). Measurements were taken before the induction of anesthesia, one and three minutes after rocuronium administration, and one, three, five, and 10 minutes after tracheal intubation. Secondary endpoints included the number of patients requiring vasopressors and vasopressor dosage, time to fall asleep, and BIS values. All values are expressed as the median (interquartile range). Continuous variables were compared using the Mann-Whitney U test. Statistically significant differences were set at p-values <0.05.

Results: Forty patients (20 in each group) were included in the final analysis. The doses of remimazolam and midazolam until sleep onset were 0.058 (0.053, 0.066) mg/kg in the R group and 0.035 (0.03, 0.045) mg/kg in the M group. The MAP at five minutes and 10 minutes after tracheal intubation was significantly higher in the R group than in the M group (p=0.031 and p=0.004, respectively). The HR, CI, SVI, SVRI, and SVV were not significantly different between the two groups at any of the measurement points. The number of patients requiring vasopressors and vasopressor dosage were not statistically significant between the two groups. The time to fall asleep was 124 seconds (90, 142) in the R group and 146 seconds (130, 167) in the M group, with a significant difference (p=0.01). The BIS values during anesthesia induction were not significantly different between the two groups.

Conclusion: Remimazolam had fewer hemodynamic effects than midazolam, even with relatively high doses and an earlier sleep onset. In terms of hemodynamic stability, remimazolam may be beneficial during anesthetic induction; however, further research is needed to confirm its efficacy.

## Introduction

The induction of general anesthesia may cause hemodynamic deterioration in patients due to the direct effects of anesthetic agents, indirect effects via sympathetic depression, and the effects of positive pressure ventilation. In particular, patients undergoing cardiovascular surgery have several cardiovascular risk factors, and anesthetic agents with minimal hemodynamic effects should be selected for their anesthesia induction.

Among the sedatives used in general anesthesia, midazolam, a classic benzodiazepine, is known to have relatively weak circulatory depression [1] and has been reported to have better hemodynamics during sedation and anesthetic induction than propofol, dexmedetomidine, and diazepam [2,3]. On the other hand, remimazolam is a new short-acting benzodiazepine general anesthetic approved in Japan in January 2020 [4]. Remimazolam has almost the same chemical structure as midazolam, except for a side chain with a carboxyl ester bond related to metabolism [4], and the sedative potency of remimazolam is equivalent to that of midazolam [5]. Therefore, the hemodynamic effects of remimazolam may be similar to those of midazolam. Remimazolam has been demonstrated to have fewer hypotensive effects than propofol [6]. However, to our knowledge, no studies have compared the hemodynamic effects of remimazolam and midazolam during general anesthesia induction.

This study aimed to compare the hemodynamic effects of remimazolam and midazolam during anesthesia induction in patients undergoing cardiovascular surgery.

## Materials and methods

Study design and patient selection

Following approval from the Institutional Review Board of Sasebo City General Hospital (SCGH) (approval number: 2021-A027), we performed this single-center retrospective and exploratory study on patients who underwent cardiovascular surgery under general anesthesia at SCGH. The study was conducted in accordance with the Code of Ethics of the World Medical Association (Declaration of Helsinki) for experiments involving humans. Owing to the retrospective study design and patient anonymity, the requirement for informed consent from individual participants was waived by the Institutional Review Board. This manuscript adheres to the STROBE guidelines for observational studies [7].

The study included adult patients who underwent cardiovascular surgery under general anesthesia at SCGH between April 2021 and November 2021. Based on the sedative agent used for anesthesia, the patients were categorized into remimazolam (R group) and midazolam (M group) groups. Patients on ventilator management prior to the induction of anesthesia, patients on circulatory agonists, patients supported by assistive circulatory devices such as intra-aortic balloon pumping or extracorporeal membrane oxygenation, patients with a history of hemodialysis, and patients who had difficulty catheterizing radial arteries were excluded.

Anesthesia management

**Figure 1:**
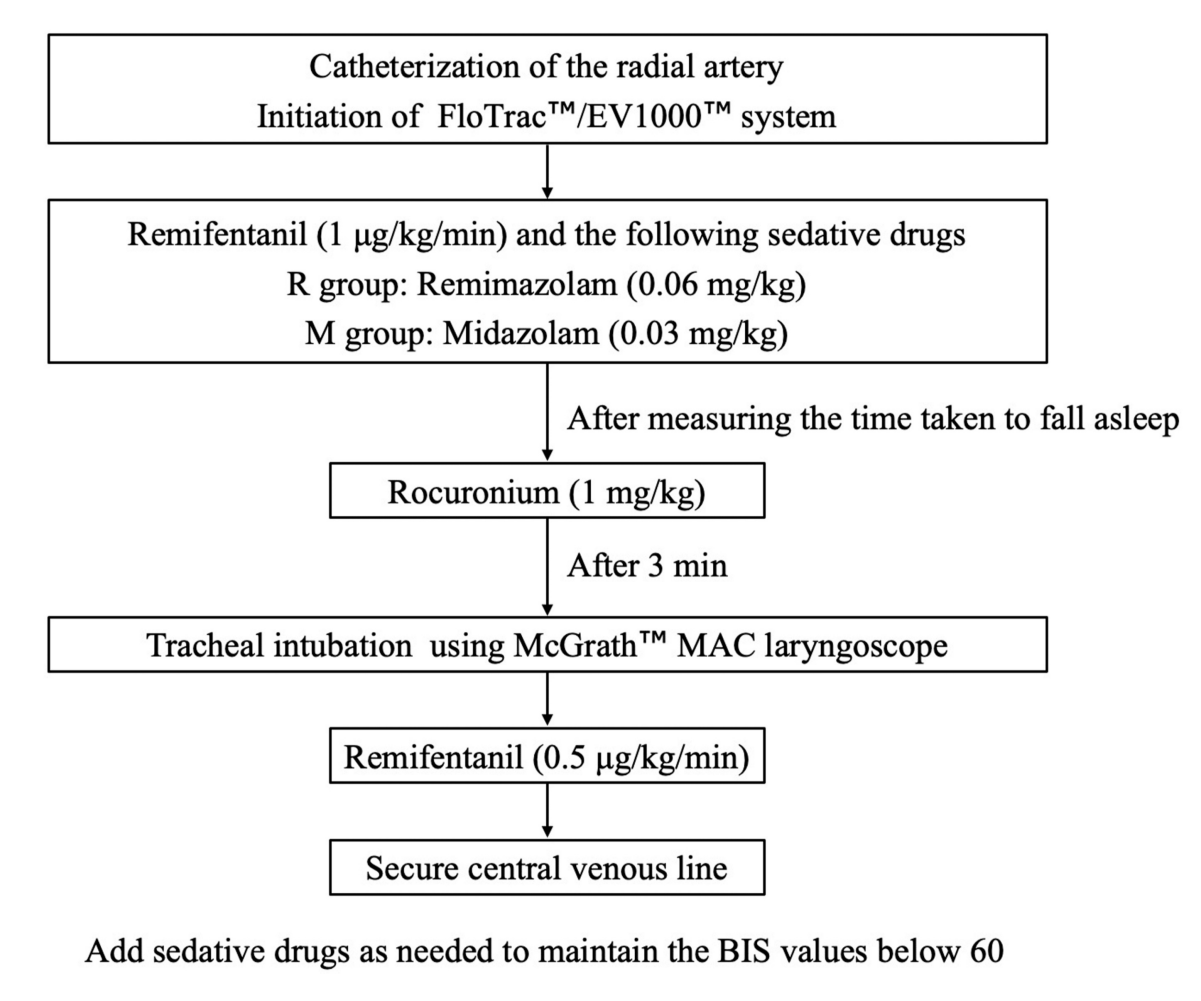
Manual for induction of anesthesia for cardiac surgery patients at Sasebo City General Hospital MAC: mean arterial pressure, BIS: bispectral index

One of the five identified anesthesiologists performed general anesthesia management. They were physicians with five to eight years of clinical experience. The SCGH has a standardized anesthesia induction procedure for cardiovascular surgery, and all anesthesiologists performed the induction as per the procedure shown in Figure 1. After placement of the radial artery catheter, remimazolam 0.06 mg/kg (R group) or midazolam 0.03 mg/kg (M group) was administered along with remifentanil 1 μg/kg/min. Time to fall asleep was measured, and 1 mg/kg of rocuronium was administered; three minutes after rocuronium administration, tracheal intubation was performed using a videolaryngoscope (McGrath^TM^ MAC; Medtronic, Dublin, Ireland). After tracheal intubation, the dose of remifentanil was reduced to 0.5 μg/kg/min and a central venous catheter was placed.

Regarding the dosage of anesthetic drugs, low-dose midazolam (0.03 mg/kg) combined with high-dose remifentanil (1 μg/kg/min) has generally been employed for anesthetic induction in patients undergoing cardiovascular surgery at our institution [8]. Additionally, since remimazolam has a shorter half-life than midazolam [4] and was administered as a relatively low single dose rather than as a continuous infusion, the dosage of remimazolam (0.06 mg/kg) for anesthetic induction was experientially set at twice the dose of midazolam. Additional sedatives were administered to maintain the bispectral index (BIS) below 60 (remimazolam or midazolam, respectively), and the additional dosage was left to the discretion of the anesthesiologist in charge. Tracheal intubation was performed three minutes after rocuronium administration because a state of complete muscle relaxation is necessary to avoid cough reflex and cardiovascular fluctuations during intubation [9]. There were no criteria for the administration of vasopressors, and the choice and combination of vasopressors was left to the discretion of the anesthesiologist in charge.

The FloTrac™/EV1000™ system (Edwards Lifesciences, Irvine, USA), a minimally invasive cardiac output monitoring by analyzing the arterial waveform, was used to analyze the hemodynamic parameters, such as cardiac index (CI), stroke volume index (SVI), systemic vascular resistance index (SVRI), and stroke volume variation (SVV). Moreover, SVRI was calculated using the following formula: (MAP - CVP) × 80/CI, and CVP was set uniformly at 5 mmHg because the central venous catheter was placed after anesthesia induction.

Data collection

All patient data were investigated using our electronic medical record system (HOPE EGMAIN™; Fujitsu, Tokyo, Japan) and anesthesia record system (Mirrel™; Fukuda Denshi, Tokyo, Japan). Baseline patient demographics, medical history, comorbidities, preoperative echocardiographic findings, and surgical and anesthetic characteristics were obtained for all study participants.

Study endpoint

The primary endpoints of the study were the following hemodynamic parameters: mean arterial pressure (MAP), HR, CI, SVI, SVRI, and SVV, which were investigated at seven points: before anesthesia induction (T0), one and three minutes after rocuronium administration (T1, 2) and one, three, five, and 10 minutes after tracheal intubation (T3, 4, 5, 6). The secondary endpoints included the number of patients requiring vasopressors and vasopressor dosage, time to fall asleep, and BIS values. In addition, residual memories during anesthesia induction were investigated as adverse events. These were defined as patient complaints during postoperative interviews.

Statistical analysis

No statistical sample size calculations were conducted. The collected data were compared between the R and M groups. The Chi-square test or Fisher's exact test was used to analyze categorical variables, and the Mann-Whitney U test was used to analyze continuous variables. All values are expressed as the median (interquartile range) or number of patients (%). All reported p-values were two-tailed, and statistically significant differences were set at p<0.05. Missing data were excluded from the analysis. All statistical analyses were performed using JMP™ Pro 16.0.0 (SAS Institute Inc., Cary, NC, USA).

## Results

**Figure 2:**
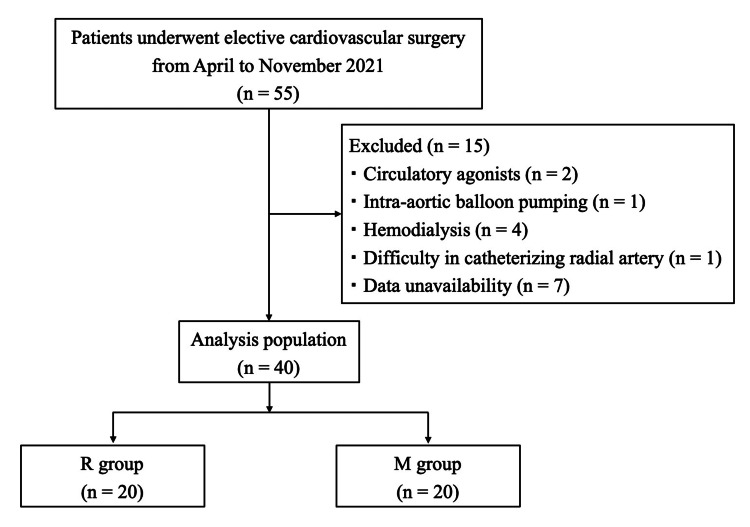
Study flow diagram detailing the selection of patients included in the retrospective analysis

Fifty-five patients underwent cardiovascular surgery during the study period, of which 15 were excluded. As a result, 40 patients were included in the final analysis: 20 in the R group and 20 in the M group (Figure 2).

Table 1 shows the baseline characteristics of patients in the R and M groups. There were no significant differences in age, sex, body mass index, or American Society of Anesthesiologists Physical Status between the two groups. There were no significant differences in preoperative cardiac function between the two groups regarding the New York Heart Association classification or transthoracic echocardiographic findings. There were no significant differences in comorbidities, medications, or type of surgery between the two groups. The doses of remimazolam and midazolam until sleep onset were 0.058 (0.053, 0.066) mg/kg in the R group and 0.035 (0.030, 0.045) mg/kg in the M group. The total dose up to 10 minutes after intubation (T6) was 0.062 (0.056, 0.081) mg/kg in the R group and 0.036 (0.031, 0.051) mg/kg in the M group.

**Table 1:** Baseline characteristics of the study population Values are presented as the median (interquartile range) or number of patients (%). Valvular disease was moderate and severe. *Others included total arch replacement, ascending aortic replacement, and aortic valve replacement. ASA-PS: American Society of Anesthesiologists, NYHA: New York Heart Association, LVDd: left ventricular end-diastolic diameter, LVEF: left ventricular ejection fraction, E/e’; E: mitral flow velocity during early diastole, e’: early diastolic wall motion velocity of the mitral valve annulus, TRPG: tricuspid regurgitant pressure gradient, AS: aortic stenosis, AR: aortic regurgitation, MS: mitral stenosis, MR: mitral regurgitation, TR: tricuspid regurgitation, COPD: chronic obstructive pulmonary disease, CABG: coronary artery bypass grafting

	R group (n = 20)	M group (n = 20)	p-value
Age (years)	73 (66, 78)	72 (69, 75)	0.797
Male	14 (70%)	13 (65%)	0.999
Body mass index (kg/m^2^)	24 (21, 26)	24 (23, 26)	0.715
ASA-PS	-	-	0.663
Class Ⅱ	7(%)	5(%)	-
Class Ⅲ	10(%)	13(%)	-
Class Ⅳ	3(%)	2(%)	-
NYHA	-	-	0.119
Class I	7 (%)	2 (%)	-
Class Ⅱ	8 (%)	7 (%)	-
Class Ⅲ	4 (%)	10 (%)	-
Class Ⅳ	1 (%)	1 (%)	-
LVDd (mm)	48 (45, 55)	50 (45, 51)	0.957
LVEF (%)	61 (48, 66)	64 (57, 69)	0.636
E/e’-septal-	13 (8, 19)	16 (12, 19)	0.250
E/e’-lateral-	10 (8, 14)	10 (8, 16)	0.425
TRPG (mmHg)	22 (20, 28)	24 (19, 30)	0.925
AS	3 (15%)	5 (25%)	0.695
AR	4 (20%)	2 (10%)	0.661
MS	0	0	-
MR	2 (10%)	6 (30%)	0.235
TR	0	2 (10%)	0.487
COPD	1 (5%)	1 (5%)	0.999
Chronic heart failure	7 (35%)	6 (30%)	0.999
Ischemic heart disease	4 (20%)	7 (35%)	0.480
Atrial fibrillation	1 (5%)	2 (10%)	0.999
Cerebrovascular disease	2 (10%)	1 (5%)	0.999
Chronic Kidney Disease	12 (60%)	11 (55%)	0.999
Liver dysfunction	0	0	-
Hypertension	17 (85%)	16 (80%)	0.999
Diabetes mellitus	7 (35%)	4 (20%)	0.479
Coronary artery dilators	2 (10%)	7 (35%)	0.127
Calcium channel blockers	12 (60%)	12 (60%)	0.999
Renin-angiotensin system inhibitors	8 (40%)	9 (45%)	0.999
Beta-blockers	8 (40%)	10 (50%)	0.750
Type of surgery	-	-	0.999
CABG	9 (45%)	9 (45%)	-
Valve	8 (40%)	8 (40%)	-
CABG + valve	0	1 (5%)	-
*Others	3 (15%)	2 (10%)	-

Primary endpoint

**Figure 3:**
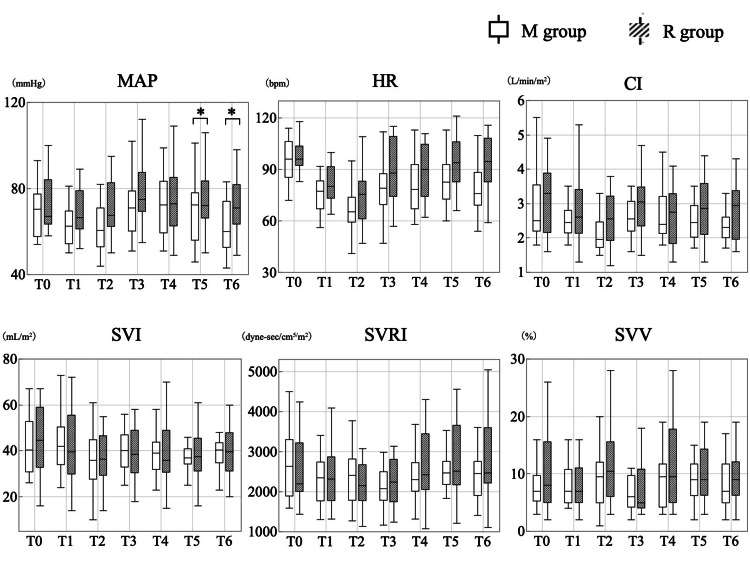
Comparison of hemodynamic parameters during anesthesia induction *Significant difference compared to the M group (p<0.05). The MAP at five (T5) and 10 minutes (T6) after tracheal intubation was significantly higher in the R group than in the M group, with no differences in other hemodynamic parameters. MAP: mean arterial pressure, HR: heart rate, CI: cardiac index, SVI: stroke volume index, SVRI: systemic vascular resistance index, SVV: stroke volume variation, R group: remimazolam group, M group: midazolam group

The hemodynamic parameters during anesthesia induction are shown in Figure 3 and Table 2. The MAP at 5 minutes (T5) and 10 minutes (T6) after tracheal intubation was significantly higher in the R group than in the M group (p=0.031 and p=0.004; T5 and T6, respectively). The HR, CI, SVI, SVRI, and SVV were not significantly different between the two groups.

**Table 2:** Summary statistics for hemodynamic parameters and BIS Values are presented as the median (interquartile range). BIS: bispectral index, MAP: mean arterial pressure, HR: heart rate, CI: cardiac index, SVI: stroke volume index, SVRI: systemic vascular resistance index, SVV: stroke volume variation, R group: remimazolam group, M group: midazolam group

	T0	T1	T2	T3	T4	T5	T6
MAP (mmHg)							
R group	96 (92, 104)	80 (73, 92)	76 (61, 83)	88 (74, 109)	90 (74, 105)	94 (83, 106)	95 (83, 108)
M group	96 (86, 107)	78 (67, 84)	66 (59, 74)	79 (70, 88)	79 (67, 93)	83 (73, 93)	76 (69, 88)
HR (bpm)							
R group	67 (63, 84)	67 (61, 79)	68 (62, 83)	75 (69, 87)	73 (63, 85)	72 (66, 84)	71 (64, 82)
M group	71 (58, 78)	63 (55, 70)	61 (53, 71)	71 (60, 79)	73 (60, 84)	73 (56, 78)	60 (53, 74)
CI (L/min/m^2^)							
R group	3.3 (2.2, 3.9)	2.6 (2.1, 3.4)	2.6 (1.9, 3.2)	3.1 (2.4, 3.5)	2.8 (1.8, 3.3)	2.9 (2.1, 3.6)	3.0 (2.0, 3.4)
M group	2.5 (2.2, 3.6)	2.5 (2.2, 2.8)	2.0 (1.7, 2.5)	2.6 (2.2, 3.1)	2.4 (2.1, 3.2)	2.5 (2.0, 3.0)	2.3 (2.0, 2.6)
SVI (mL/m^2^)							
R group	45 (33, 59)	40 (30, 56)	37 (29, 47)	39 (30, 49)	36 (31, 49)	38 (31, 46)	40 (31, 48)
M group	41 (31, 53)	42 (34, 51)	36 (28, 45)	40 (33, 47)	39 (32, 44)	37 (34, 41)	41 (35, 44)
SVRI (dyne-sec/cm^5^/m^2^)							
R group	2201 (1996, 3215)	2318 (1766, 2857)	2153 (1771, 2668)	2249 (1739, 2800)	2431 (2044, 3436)	2519 (2163, 3654)	2463 (2205, 3584)
M group	2630 (1899, 3306)	2340 (1778, 2744)	2413 (1782, 2819)	2081 (1792, 2494)	2308 (2009, 2724)	2472 (2180, 2751)	2457 (1911, 2756)
SVV (%)							
R group	8 (5, 16)	7 (5, 11)	11 (6, 16)	5 (4, 11)	10 (5, 18)	9 (6, 14)	9 (6, 12)
M group	7 (5, 10)	7 (5, 11)	10 (5, 12)	6 (4, 10)	10 (4, 12)	9 (6, 12)	7 (5, 12)
BIS							
R group	97 (90, 97)	76 (63, 85)	45 (39, 48)	41 (34, 46)	38 (32, 43)	42 (33, 45)	45 (36, 49)
M group	96 (93, 97)	65 (48, 78)	42 (36, 46)	41 (38, 44)	41 (37, 44)	42 (38, 51)	47 (44, 57)

Secondary endpoint

**Figure 4:**
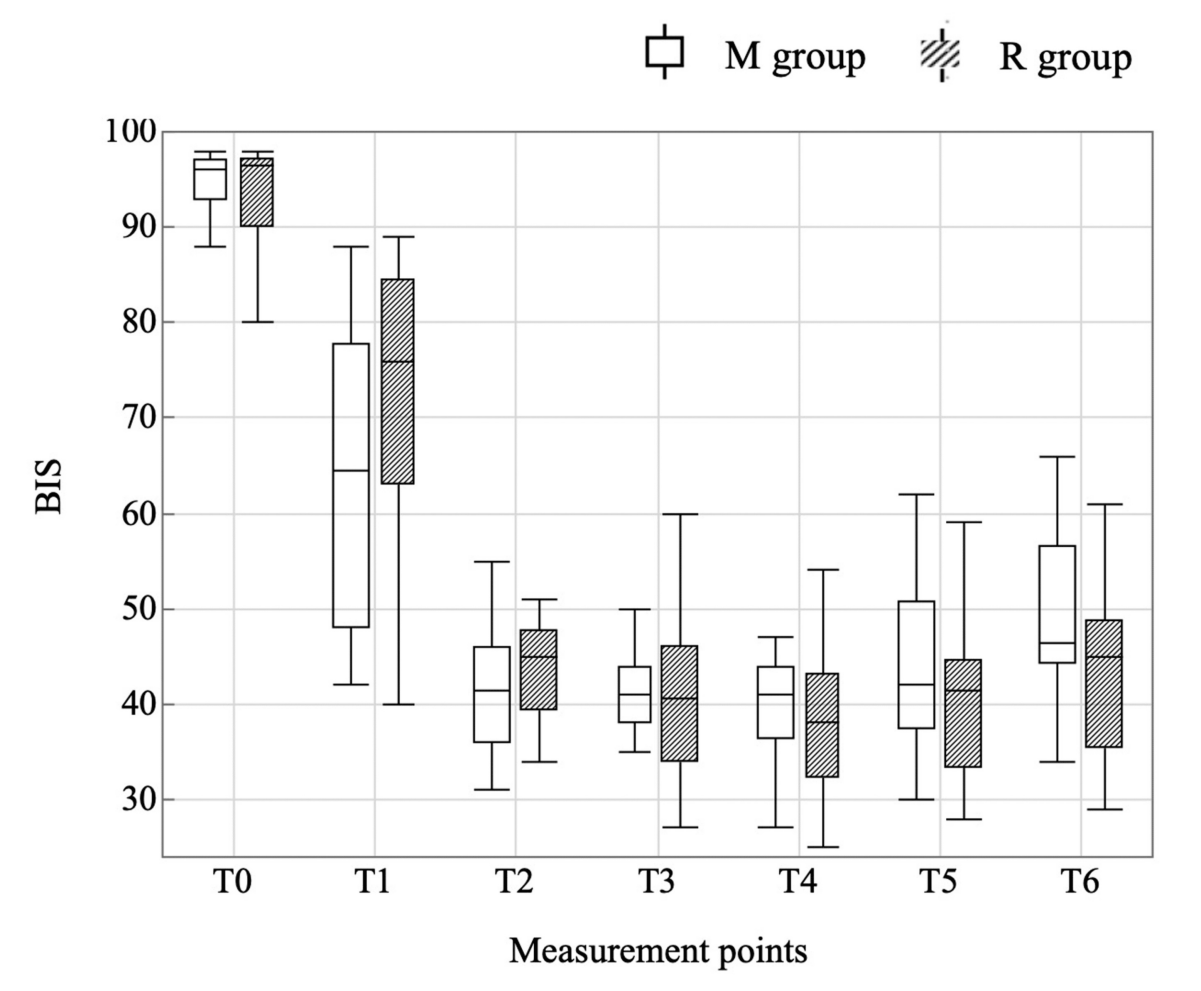
BIS values during anesthesia induction BIS: bispectral index

The number of patients who required vasopressors was 3 (15%) in the R group and 5 (25%) in the M group, with no statistically significant difference (p=0.695). Phenylephrine was the vasopressor of choice for all patients. The dose was 0 (0, 0) mg in the R group and 0 (0, 0.075) mg in the M group, with no statistically significant difference (p=0.45). The time required to fall asleep was 124 seconds (90, 142) in the R group and 146 seconds (130, 167) in the M group, significantly faster in the R group (p=0.005). The BIS values during anesthesia induction were not significantly different between the two groups at any of the measurement points (Figure 4).

Adverse event

No patient complained of having residual memories of the anesthesia induction.

## Discussion

This retrospective study is the first report comparing the hemodynamic effects of remimazolam and midazolam during induction of general anesthesia. The MAP after tracheal intubation was higher in patients treated with remimazolam than in those treated with midazolam, although the BIS values were comparable. Additionally, patients treated with remimazolam fell asleep faster than those who received midazolam, which had less effect on hemodynamic parameters.

In this study, the MAP from the start of anesthesia induction to three (T4) minutes after intubation was not different between the two groups; however, the MAP in the R group at five (T5) and 10 (T6) minutes after intubation was significantly higher than that in the M group. Although the sedative potency is almost the same between remimazolam and midazolam [5], the systemic clearance of remimazolam is approximately three times that of midazolam, and the volume of distribution is less than half that of midazolam [10]. The median time to return to a fully awake state after the sedation of a participant with 0.075 mg/kg of remimazolam or midazolam is 5.5 minutes for remimazolam compared to 40 minutes for midazolam [10]. Therefore, one reason for the difference in MAP after intubation may be the brief effect of remimazolam compared to that of midazolam.

Another reason is that remimazolam may have fewer hemodynamic effects than midazolam. In this study, sleep onset was significantly faster in the R group than in the M group, likely because the induction dose of remimazolam was approximately 1.7 times higher than that of midazolam (0.058 mg/kg vs. 0.035 mg/kg; R group and M group, respectively). However, despite the higher dose, the MAP before intubation in the R group was similar to that in the M group. In a study comparing the remimazolam and midazolam sedation during colonoscopy, 10 mg of remimazolam and 4 mg of midazolam were administered in addition to approximately 100 μg of fentanyl; the degree of sedation assessed by the MOAA/S score was deeper in the R group, but the incidence of hypotension (defined as systolic blood pressure ≦80 mmHg) was lower in the R group (38.9%) than in the M group (61.8%) [11]. Although this differs from general anesthesia induction, the data support our findings.

The clinical relevance of this study is that remimazolam may be useful in maintaining MAP during the period after tracheal intubation to the start of surgery when the hemodynamics are unstable due to less nociceptive stimulation. Additionally, the induction doses of remimazolam and midazolam in this study were relatively low compared to typical doses because they were combined with high-dose remifentanil. However, even at these low doses, remimazolam still had less effect on hemodynamic parameters under the same anesthetic induction protocol, except for these two drugs, and patients who received remimazolam fell asleep faster than those who received midazolam. Therefore, although further studies are necessary, at typical doses for anesthetic induction, remimazolam may have much less hemodynamic instability than midazolam.

The present study had several limitations. First, it was a single-center, backward-looking exploratory study with a small sample size; therefore, it may be difficult to generalize the results obtained in this study. However, the SCGH used a standardized protocol for the induction of anesthesia in patients undergoing cardiovascular surgery, and factors other than remimazolam and midazolam remained constant. Thus, the present results may reflect the differences in the effects of the two drugs on hemodynamics. Second, although the BIS values between the two groups did not differ in this study, the actual sedation levels may not have been comparable. Evaluating sedation levels by comparing the hemodynamic effects of two anesthetic drugs is important. However, remifentanil slows the EEG when blood levels exceed 10 ng/mL (approximately 0.4 μg/kg/min as a continuous dose) [12]. For this reason, the high-dose remifentanil (around 1.0 μg/kg/min) used in this study may have affected BIS values and not reflected actual sedation levels. However, in another study, remifentanil had no dose-dependent effect on BIS value at 0, 0.5, and 1.0 μg/kg/min [13]. In addition, the M group took 22 seconds (median) longer to fall asleep than the R group, which means that remifentanil was also administered longer in the M group. Therefore, it cannot be ruled out that the lower blood pressure at T5 and T6 in the M group may have been caused by a high plasma concentration of remifentanil. Finally, in addition to blood pressure and heart rate, more detailed hemodynamic parameters, such as CI, SVI, SVRI, and SVV, were analyzed in this study using the FloTrac™/EV1000™ system. Although MAP after intubation was significantly higher in the R group than in the M group, none of the other hemodynamic parameters differed between the two groups. This is probably because the FloTrac™/EV1000™ system generally cannot follow rapid hemodynamic changes [14,15]. Moreover, the SVRI [= (MAP - CVP) × 80/CI] may be unreliable because it was calculated assuming a uniform CVP of 5 mmHg.

## Conclusions

This study compared the hemodynamic effects of remimazolam and midazolam during anesthesia induction in patients undergoing cardiovascular surgery. Remimazolam had fewer hemodynamic effects than midazolam, even with relatively high doses and an earlier sleep onset. In terms of hemodynamic stability, remimazolam may be beneficial during anesthetic induction; however, further research is needed to confirm its efficacy.

## References

[1] Kanto JH (1985). Midazolam: the first water-soluble benzodiazepine. Pharmacology, pharmacokinetics and efficacy in insomnia and anesthesia. Pharmacotherapy.

[2] Frölich MA, Arabshahi A, Katholi C, Prasain J, Barnes S (2011). Hemodynamic characteristics of midazolam, propofol, and dexmedetomidine in healthy volunteers. J Clin Anesth.

[3] Kanaya N, Fujita S, Tsuchida H, Seki S, Namiki A (1994). The effects of low-dose midazolam for induction of high-dose fentanyl anesthesia for coronary artery bypass graft. J Anesth.

[4] Masui K (2020). Remimazolam besilate, a benzodiazepine, has been approved for general anesthesia!!. J Anesth.

[5] Kilpatrick GJ, McIntyre MS, Cox RF (2007). CNS 7056: a novel ultra-short-acting benzodiazepine. Anesthesiology.

[6] Doi M, Morita K, Takeda J, Sakamoto A, Yamakage M, Suzuki T (2020). Efficacy and safety of remimazolam versus propofol for general anesthesia: a multicenter, single-blind, randomized, parallel-group, phase IIb/III trial. J Anesth.

[7] von Elm E, Altman DG, Egger M, Pocock SJ, Gøtzsche PC, JP Vandenbroucke (2007). The strengthening the reporting of observational studies in epidemiology (STROBE) statement: Guidelines for reporting observational studies. Lancet.

[8] Kaneko S, Morimoto T, Ichinomiya T, Murata H, Yoshitomi O, Hara T (2023). Effect of remimazolam on the incidence of delirium after transcatheter aortic valve implantation under general anesthesia: a retrospective exploratory study. J Anesth.

[9] Kirkegaard-Nielsen H, Caldwell JE, Berry PD (1999). Rapid tracheal intubation with rocuronium: a probability approach to determining dose. Anesthesiology.

[10] Antonik LJ, Goldwater DR, Kilpatrick GJ, Tilbrook GS, Borkett KM (2012). A placebo- and midazolam-controlled phase I single ascending-dose study evaluating the safety, pharmacokinetics, and pharmacodynamics of remimazolam (CNS 7056): part I. Safety, efficacy, and basic pharmacokinetics. Anesth Analg.

[11] Rex DK, Bhandari R, Desta T (2018). A phase III study evaluating the efficacy and safety of remimazolam (CNS 7056) compared with placebo and midazolam in patients undergoing colonoscopy. Gastrointest Endosc.

[12] Egan TD, Muir K, Hermann D, Stanski DR, Shafer SL (2001). The electroencephalogram (EEG)and clinical measures of opioid potency: defining the EEG-clinical potency relationship (‘fingerprint’) with application to remifentanil. Int J Pharm Med.

[13] Yufune S, Takamatsu I, Masui K, Kazama T (2011). Effect of remifentanil on plasma propofol concentration and bispectral index during propofol anaesthesia. Br J Anaesth.

[14] Lee M, Weinberg L, Pearce B (2017). Agreement in hemodynamic monitoring during orthotopic liver transplantation: a comparison of FloTrac/Vigileo at two monitoring sites with pulmonary artery catheter thermodilution. J Clin Monit Comput.

[15] Maeda T, Hattori K, Sumiyoshi M, Kanazawa H, Ohnishi Y (2018). Accuracy and trending ability of the fourth-generation FloTrac/Vigileo System™ in patients undergoing abdominal aortic aneurysm surgery. J Anesth.

